# Electrodeposition of platinum and silver into chemically modified microporous silicon electrodes

**DOI:** 10.1186/1556-276X-7-330

**Published:** 2012-06-21

**Authors:** Ryo Koda, Kazuhiro Fukami, Tetsuo Sakka, Yukio H Ogata

**Affiliations:** 1 Institute of Advanced Energy, Kyoto University, Uji, Kyoto, 611-0011, Japan

**Keywords:** microporous silicon, noble metal, electrodeposition, hydration property

## Abstract

Electrodeposition of platinum and silver into hydrophobic and hydrophilic microporous silicon layers was investigated using chemically modified microporous silicon electrodes. Hydrophobic microporous silicon enhanced the electrodeposition of platinum in the porous layer. Meanwhile, hydrophilic one showed that platinum was hardly deposited within the porous layer, and a film of platinum on the top of the porous layer was observed. On the other hand, the electrodeposition of silver showed similar deposition behavior between these two chemically modified electrodes. It was also found that the electrodeposition of silver started at the pore opening and grew toward the pore bottom, while a uniform deposition from the pore bottom was observed in platinum electrodeposition. These electrodeposition behaviors are explained on the basis of the both effects, the difference in overpotential for metal deposition on silicon and on the deposited metal, and displacement deposition rate of metal.

## Background

Porous silicon has some properties such as high specific surface area and semiconductivity which allow metal electrodeposition within the porous layer without special treatment. Such properties are advantageous to the preparation of metal/semiconductor hybrid materials and catalyst-dispersed porous structures. However, there is a difficulty in metal electrodeposition within porous silicon that plugging often occurs before it is fully and/or homogeneously filled with metal [1-4]. Inhibition of plugging during metal deposition is indispensable for controlling the filling of metal by electrodeposition. For macroporous silicon, it was reported that the plugging often occurred in some metal deposition systems due to a high rate of displacement deposition which preferentially occurred at the pore opening and electrodeposition preferentially proceeded on the deposits [5]. Such an effect is not observed in the case of microporous silicon having hydrophobic pore wall, although the diameter of microporous silicon is much smaller than that of macroporous silicon. Another mechanism, which is different from the case of macroporous silicon, seems to control the deposition behavior in microporous silicon. Recently, we have found that the hydrophobic and hydrophilic properties of microporous silicon have strong effects on the platinum electrodeposition [6]. Only the hydrophobic microporous silicon accelerates the platinum deposition in the porous layer. Considering the surface hydration effect of platinum complex ions in the porous layer could explain this behavior.

In this study, electrodeposition behaviors of platinum and silver in chemically modified hydrophobic and hydrophilic microporous silicon are compared. The hydrophobic property of microporous silicon has a strong effect on platinum electrodeposition, while the silver electrodeposition is not affected by the hydrophobic and hydrophilic properties. The growth modes of the deposits show a clear difference between platinum and silver. These behaviors are explained on the basis of the both effects, the difference in overpotential for platinum and silver electrodeposition on silicon, and the difference in displacement deposition rate between platinum and silver.

## Methods

### Sample preparation

Two types of microporous silicon having various pore depths were prepared by anodization. *p*-type silicon (100) with a resistivity of 10 to 20 Ω cm was used. A mixture of 22 wt.% hydrogen fluoride/ethanol solution (47 wt.% hydrogen fluoride aqueous solution:ethanol = 1:1.7 in volume) was used for the electrolyte. Anodization was carried out for 20 and 60 min at 2 mA cm^−2^. The depth of microporous structure was ca. 2 and 7 μm, which was controlled by the duration of anodization. When the sample was anodized for 60 min under this condition, the microporous structure showed a macropore-like structure filled with microporous silicon [7]. We call this structure ‘skeleton structure.’ After anodization, microporous silicon substrates were immersed for 15 h in *n*-hexane containing 0.2 M methyl propiolate and 0.2 M propiolic acid to make the pore wall hydrophobic and hydrophilic by hydrosilylation reaction, respectively [8].

Platinum and silver were electrodeposited on the chemically modified microporous silicon electrodes. A platinum rod was used as the counter electrode. Platinum electrodeposition was carried out using 0.1 M K_2_PtCl_4_ + 0.5 M NaCl solution, and silver electrodeposition was carried out using 0.1 M AgNO_3_ + 0.5 M KNO_3_ solution. Current density for the electrodeposition was set at a constant value of−6.4 μA cm^−2^ in all the cases.

### Characterization

Current density-potential (*i-E*) curves were measured. A platinum plate, bare *p*-type silicon, and chemically modified *p*-type silicon were used as the working electrodes, where plain silicon was employed without porosification. The counter electrode was a platinum rod, and the reference electrode was an Ag/AgCl saturated KCl electrode. Scan rate of potential was set at 10 mV s^−1^.

A scanning electron microscope (JSM-6500 F, JEOL Ltd., Tokyo, Japan) was employed to obtain secondary electron images (SEI) and back scattered electron images (BEI) of the cross-sectional microporous silicon after metal electrodeposition. SEI illustrates the morphology. On the other hand, BEI provides atomic number contrast: platinum and silver deposits appear brighter than silicon in the images.

## Results

### Characterization of platinum and silver bath

**Figure 1:**
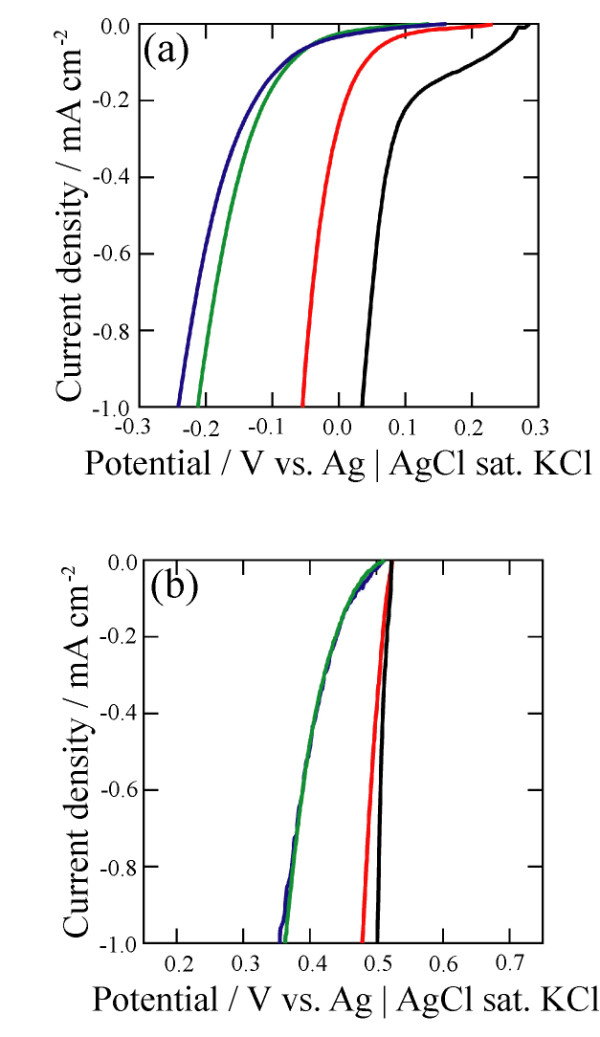
**Current density-potential curves measured with various electrodes.** The black, red, blue, and green curves were measured with a platinum or silver plate, bare *p*-type silicon, *p*-type silicon modified by propiolic acid, and *p*-type silicon modified by methyl propiolate, respectively. The scan rate of potential was 10 mV s^−1^. (**a**) 0.1 M K_2_PtCl_4_ + 0.5 M NaCl, (**b**) 0.1 M AgNO_3_ + 0.5 M KNO_3_.

*i-E* curves were measured to understand the electrodeposition behavior of the platinum and silver baths on various electrodes. Figure 1 indicates that the *i-E* curves measured with flat silicon modified by the propiolic acid and methyl propiolate in both solutions are similar in shape. These results show that the difference in overpotential between the chemically modified silicon electrodes is not large for platinum electrodeposition. Compared to the platinum plate, the *i-E* curves of the chemically modified flat silicon shift to negative potential in the platinum bath (Figure 1a). As a result, higher overpotential is necessary at the chemically modified silicon compared with the platinum plate.

The *i-E* curves measured in silver bath behave at two differently modified silicon electrodes like the platinum case (Figure 1b). However, unlike the platinum case, a shift to negative potential is very small at the silicon electrodes. The result suggests that, in case of silver, the silicon electrodes do not require a higher overpotential than in the case of platinum.

### Metal electrodeposition in chemically modified microporous silicon

**Figure 2:**
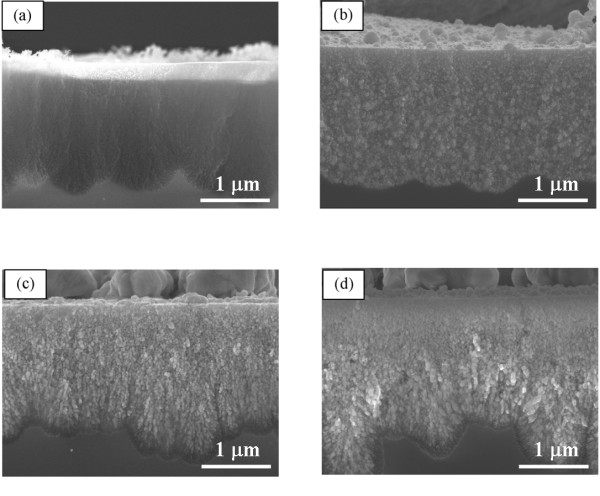
**Cross-sectional SEI of microporous silicon after the electrodeposition of platinum and silver.** After the electrodeposition of platinum and silver. Images of (**a**) and (**b**) are the cross-sections of platinum deposition in 0.1 M K_2_PtCl_4_ + 0.5 M NaCl, and those of (**c**) and (**d**) are case of silver deposition in 0.1 M AgNO_3_ + 0.5 M KNO_3_. The images in (a, c) and (b, d) show the samples of the hydrophilic and hydrophobic microporous silicon, respectively.

Platinum and silver electrodeposition was carried out for chemically modified microporous silicon with the depth of 2 μm. The cross-sectional images of microporous silicon after electrodeposition of platinum are shown in Figure 2a,b. When the pore wall is hydrophilic, platinum is deposited only on the top of microporous silicon. On the other hand, when the pore wall is hydrophobic, platinum is deposited inside microporous silicon as reported elsewhere [6].

However, when silver electrodeposition was carried out, deposition proceeds uniformly both in the chemically modified hydrophobic and hydrophilic microporous silicon (Figure 2c,d). Electrodeposition of silver is hard to be affected by the hydration properties of pore wall, such as hydrophobic or hydrophilic.

### Time development of silver electrodeposition

**Figure 3:**
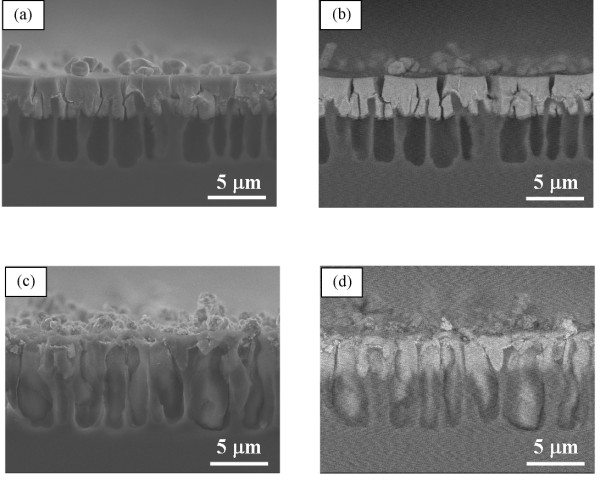
**Cross-sectional views of microporous silicon after the electrodeposition of silver for 1 min.** Images of (**a**, **b**) and (**c**, **d**) show the samples of hydrophilic and hydrophobic microporous silicon substrates, respectively. The images in (**a**) and (**c**) illustrate the SEI, and (**b**) and (**d**) illustrate the BEI.

**Figure 4:**
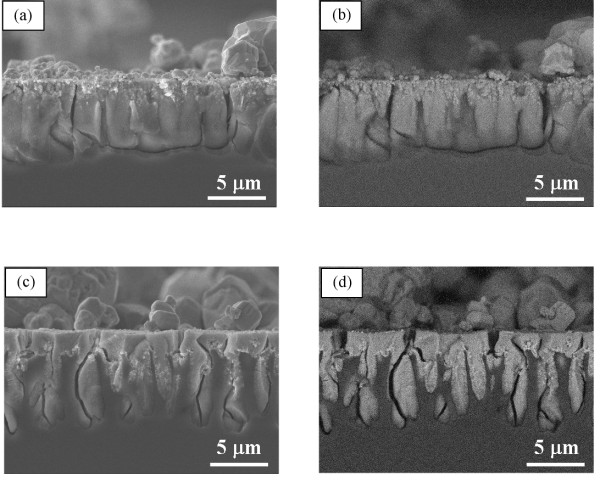
**Cross-sectional views of microporous silicon after the electrodeposition of silver for 2 min.** Images of (**a**, **b**) and (**c**, **d**) show the samples of hydrophilic and hydrophobic microporous silicon, respectively. The images in (**a**) and (**c**) illustrate the SEI, and (**b**) and (**d**) illustrate the BEI.

To investigate the reason why the hydrophobic and hydrophilic properties of microporous silicon do not affect the electrodeposition behavior of silver, time development of silver electrodeposition in a chemically modified hydrophobic microporous silicon with the pore depth of ca. 7 μm was studied. Figures 3 and 4 show cross-sectional views of the samples after electrodeposition for 1 and 2 min, respectively. After electrodeposition for 1 min, silver is deposited from the pore opening to the middle depth of the pore, which is independent of hydrophobic and hydrophilic properties. After 2-min electrodeposition, silver is uniformly deposited in both microporous silicon. The uniform deposition is also independent of hydrophobic and hydrophilic properties. These results show that silver deposition proceeds from the pore opening to the pore bottom, and these phenomena are independent of the hydration properties of pore wall, such as hydrophobic or hydrophilic.

## Discussion

### Difference in metal growth direction between platinum and silver

Current mainly flows from the pore bottom when the electrodeposition is carried out on the porous silicon substrate. In addition, the resistivity of the microporous part is high. From these points of view, metal deposition should occur from the direction of the pore bottom to the opening. In fact, platinum deposition starts at the pore bottom of microporous structure in the present case. However, when silver electrodeposition is carried out on microporous silicon substrate, such growth does not appear, and silver deposition proceeds from the pore opening to the pore bottom (Figures 3 and 4). As discussed below, the effect of displacement deposition occurring during electrodeposition and the different polarization behaviors on silicon and deposited metal can explain the difference in the growth direction.

Electrodeposition is often accompanied by displacement deposition during noble metal electrodeposition in porous silicon. In case of platinum, the electrodeposition is prevailing compared to displacement deposition because the rate of platinum displacement deposition on silicon substrate is slow [4]. Pore bottom is more favorable to electrodeposition than the opening in highly resistive microporous silicon, as mentioned above; hence, the deposition proceeds from the direction of the pore bottom to the opening. This type of growth is observed for the hydrophobic porous silicon surface.

Figure 1a shows that platinum deposition on a silicon electrode needs much higher overpotential than that on a platinum electrode. Supposing that platinum deposition occurs on the top surface, the reaction preferentially proceeds on the top, and platinum is not deposited inside the porous layer. In addition, it was reported that, when the nucleation of metal starts at the pore opening in the initial stage of the metal electrodeposition, the metal is preferentially deposited there, and the pore opening is plugged soon [1,2]. This type of growth is observed in porous silicon with the hydrophilic surface.

On the other hand, in case of silver, displacement deposition rate is extremely fast on silicon. Therefore, even though electrodeposition is carried out, displacement deposition proceeds preferentially compared to electrodeposition. It was reported that metal displacement deposition proceeds from the pore opening to the pore bottom in the macroporous structure [3]. Thus, silver electrodeposition also proceeds from the pore opening to the pore bottom in microporous structure because the effect of displacement deposition is stronger than that of electrodeposition. In addition, the potentials of silver deposition on the silicon electrode and silver electrode are similar (Figure 1b). These results show that, in the silver system, overpotential difference for silver electrodeposition on the silicon electrode and deposited silver is very small. Silver is deposited with almost equal rate on silver nuclei and bare silicon surface. The reaction does not preferentially proceed on the deposited surface at the pore opening. Therefore, pore opening is hard to be plugged, and silver ions can diffuse into the porous structure.

### Why is silver not affected by property of the pore wall?

Platinum is deposited within the hydrophobic microporous silicon in Figure 2. However, when the pore wall is hydrophilic, platinum deposition occurs only on the top of the porous layer. These results can be explained by the affinity of the pore wall to water molecules. Platinum complex ion ([PtCl_4_^2−^) behaves like a molecule with lower polarity due to its large size. Therefore, platinum ions have high affinity to the hydrophobic pore wall compared with water molecules to the pore wall, and hence, platinum ions are excluded from bulk to the pore wall [6]. In case of platinum, platinum ions pass through a bare silicon surface because deposition proceeds toward the pore opening from the pore bottom. Therefore, platinum deposition behavior is affected by the hydration property of pore wall. In hydrophilic microporous silicon, once deposition occurs at the pore opening, platinum is not deposited into pores but on the top surface due to the need of high overpotential on the bare silicon.

However, silver deposition is not affected by the hydration properties of the pore wall. These results can be explained by the growth direction of deposition. Metal ions have to reach to the frontier of the deposition site and pass through not a bare silicon surface but the surface on which metal is deposited when metal deposition proceeds toward the pore bottom. Once the metal is deposited on the pore wall, the hydration property of pore wall changes due to the metal covering. Therefore, silver is uniformly deposited in microporous layer without relation to the hydration properties of the pore wall.

## Conclusions

When the pore diameter of porous silicon is extremely small, platinum deposition is strongly affected by the hydration property of pore wall. On the other hand, silver deposition is not affected by the property. The results could be explained by the difference in overpotential between the deposition on the metal surface and that on the silicon surface, and by the difference in intrinsic displacement deposition rate of metal. When metal electrodeposition is carried out in microporous silicon, the effect of the overpotential and the displacement deposition rate should be considered together with the hydration property of the metal ions.

## Competing interests

The authors declare that they have no competing interests.

## Authors’ contributions

RK and KF designed the experiments. RK carried out all the experimental work. TS and YHO supervised the work and joined the discussion. RK prepared the first draft of the manuscript. All authors read, revised, and approved the final manuscript.

## Authors’ information

RK is a PhD student in the Institute of Advanced Energy, Kyoto University. KF, TS and YHO are professors of the same university.
